# Cardiovascular Risk Biomarkers in Women with and Without Polycystic Ovary Syndrome

**DOI:** 10.3390/biom15010004

**Published:** 2024-12-24

**Authors:** Manjula Nandakumar, Priya Das, Thozhukat Sathyapalan, Alexandra E. Butler, Stephen L. Atkin

**Affiliations:** 1 Research Department, Royal College of Surgeons of Ireland, Adliya, Busaiteen 15503, Bahrain; mnandakumar@rcsi.com (M.N.); pdas@rcsi.com (P.D.); satkin@rcsi.com (S.L.A.); 2 Academic Endocrinology, Diabetes and Metabolism, Hull York Medical School, Hull HU6 7RU, UK; thozhukat.sathyapalan@hyms.ac.uk

**Keywords:** polycystic ovary syndrome, PCOS, cardiovascular risk, biomarkers, proteomics

## Abstract

Objective: Polycystic ovary syndrome (PCOS) is a prevalent metabolic disorder with an increased risk for cardiovascular disease (CVD) that is enhanced by obesity. This study sought to determine whether a panel of cardiovascular risk proteins (CVRPs) would be dysregulated in overweight/obese PCOS patients, highlighting potential biomarkers for CVD in PCOS. Methods: In this exploratory cross-sectional study, plasma levels of 54 CVRPs were analyzed in women with PCOS (n = 147) and controls (n = 97). CVRPs were measured using the SOMAscan proteomic platform (version 3.1), with significant proteins identified through linear models, regression analysis, and receiver operating characteristic (ROC) analysis. Analysis on BMI-matched subsets of the cohort were undertaken. Functional enrichment and protein–protein interaction analyses elucidated the pathways involved. Results: Eleven CVRPs were dysregulated in PCOS (whole set, without matching for body mass index (BMI) or insulin resistance (IR)): leptin, Interleukin-1 receptor antagonist protein (IL-1Ra), polymeric immunoglobulin receptor (PIGR), interleukin-18 receptor (IL-18Ra), C-C motif chemokine 3 (MIP-1a), and angiopoietin-1 (ANGPT1) were upregulated whilst advanced glycosylation end product-specific receptor, soluble (sRAGE), bone morphogenetic protein 6 (BMP6); growth/differentiation factor 2 (GDF2), superoxide dismutase [Mn] mitochondrial (MnSOD), and SLAM family member 5 (SLAF5) were downregulated versus the controls. In BMI-matched (overweight/obese, BMI ≥ 26 kg/m^2^) subset analysis, six CVRPs were common to the whole set: ANGPT1 and IL-1Ra were upregulated; and sRAGE, BMP6, GDF2, and Mn-SOD were downregulated. In addition, lymphotactin (XCL1) was upregulated and placenta growth factor (PIGF), alpha-L-iduronidase (IDUA), angiopoietin-1 receptor, and soluble (sTie-2) and macrophage metalloelastase (MMP12) were downregulated. A subset analysis of BMI-matched plus insulin resistance (IR)-matched women revealed only upregulation of tissue factor (TF) and renin in PCOS, potentially serving as biomarkers for cardiovascular risk in overweight/obese women with PCOS. Conclusions: A combination of upregulated obesity-related CVRPs (ANGPT1/IL/1Ra/XCL1) and downregulated cardioprotective proteins (sRAGE/BMP6/Mn-SOD/GDF2) in overweight/obese PCOS women may contribute to the increased risk for CVD. TF and renin upregulation observed in the BMI- and IR-matched limited sample PCOS subgroup indicates their potential risk of CVD.

## 1. Introduction

Polycystic ovary syndrome (PCOS) is the most prevalent metabolic disorder in reproductive women, affecting 5–10% of women [1]. Despite the establishment of international criteria for diagnosing PCOS, approximately 70% of women with the syndrome remain undiagnosed [2]. As a metabolic disorder, PCOS is associated with a higher prevalence of comorbidities such as hypertension, dyslipidemia, type 2 diabetes, and increased cardiovascular risk, underscoring its clinical importance [3]. The etiology of PCOS is multifactorial, involving a complex interplay of genetic, environmental, and lifestyle factors that contribute to its pathogenesis. The Rotterdam diagnostic criteria require two of the three features, namely biochemical or clinical hyperandrogenism, irregular periods of 10 or less per year (ovulatory dysfunction), and polycystic ovarian morphology on ultrasound. Over 30% of women with PCOS have impaired glucose regulation and up to 10% develop diabetes [4]. Mechanistically, PCOS affects reproductive, cardiovascular, and metabolic systems. Key factors such as hyperandrogenemia, chronic inflammation, oxidative stress, and insulin resistance (IR) are present in PCOS and play critical roles in the dysregulation of several cellular biomarkers such as heat shock proteins, complement proteins, and coagulation markers, largely driven by underlying obesity and IR. These mechanisms contribute to the development of systemic complications and further emphasize the need for early diagnosis and comprehensive management of PCOS. Obesity is reported in 50% of women with PCOS [5] and significantly impacts PCOS phenotypes and fertility outcomes [6].

The interplay between obesity, IR, and PCOS creates a vicious cycle that complicates metabolic and reproductive health in affected individuals. Both obesity and PCOS are linked to various diseases of the cardiovascular system such cardiovascular events [7], stroke, hypertension, and venous thromboembolism [8]. PCOS is most strongly associated with IR, which is the major underlying factor in the development of various cardiometabolic diseases such as dyslipidemia and hypertension. High circulatory levels of C-reactive protein, a marker of inflammation, as well as increased thickness and calcification of coronary arteries were associated with IR, obesity, and PCOS as subclinical diagnostic markers for cardiovascular diseases (CVDs). Although the underlying pathophysiology of PCOS causes an increase in CVD risk [7], the association of PCOS with subclinical markers of CVDs has not been well explored [9]

Understanding the etiology and systemic effects of obesity and PCOS is crucial for developing therapeutic strategies to prevent CVDs in women. In a recent study of nonobese PCOS women, nine cardiovascular risk proteins (CVRPs) were upregulated compared to women without PCOS [10]. In this study, we compare CVRP expression in women with and without PCOS, irrespective of BMI or insulin resistance, to identify PCOS-specific differences. Among these, we hypothesized that overweight/obese women with PCOS are at a higher risk of CVDs potentially reflected in a comparable or more pronounced CVRP expression profile compared to their non-PCOS counterparts.

## 2. Materials and Methods

### 2.1. Study Design

In this exploratory cross-sectional analysis, plasma levels of CVRPs were measured in Caucasian women with PCOS (n = 147) and non-PCOS (n = 97) recruited from the Hull endocrine clinic [11]. Non-PCOS women, who were recruited by advert, were age matched to the PCOS patients, and all were recruited from the same geographic region and with lower socioeconomic status. For the diagnosis of PCOS, the following Rotterdam consensus criteria were used: (1) clinical (Ferriman–Gallwey score of >8) and biochemical hyperandrogenemia (a free androgen index (FAI) of >4); (2) oligomenorrhea or amenorrhea; and (3) polycystic ovaries seen on transvaginal ultrasound [12]. Study participants had no other condition or illness and were required to be medication-free for nine months preceding study enrolment, including the exclusion of over-the-counter medication. Testing was undertaken to ensure that no patient had any of the following endocrine conditions: non-classical 21-hydroxylase deficiency, hyperprolactinemia, Cushing’s disease, or an androgen-secreting tumor as per the recommendations [13]. Demographic data for both non-PCOS and PCOS women is shown in Table 1. The study was conducted in accordance with the Declaration of Helsinki and approved by the Newcastle and North Tyneside Ethics Committee (reference number 10/H0906/17 and date of approval of 6 June 2014).

Patients presented after fasting overnight; height, weight, waist circumference, and body mass index (BMI) were recorded according to the World Health Organization (WHO) guidelines [14]. BMI was defined as weight in kilograms and height in centimeters, with the formula kg/m^2^. The participants with a BMI ranging from 26 to 29.9 kg/m^2^ were considered overweight and a BMI ≥ 30 kg/m^2^ were considered obese. Blood was withdrawn during fasting and the plasma was prepared by centrifugation at 3500· *g* for 15 min, aliquoted, and stored at −80 °C. An analysis for sex hormone binding globulin (SHBG), insulin (DPC Immulite 200 analyser, Euro/DPC, Llanberis UK), and plasma glucose (to calculate homeostasis model assessment–insulin resistance (HOMA-IR)) (Synchron LX20 analyser, Beckman-Coulter, High Wycombe, UK) was undertaken. Free androgen index (FAI) was derived from total testosterone divided by SHBG x100. Insulin resistance (IR) was determined by HOMA-IR (insulin × glucose)/22.5). Serum testosterone was quantified using isotope-dilution liquid chromatography tandem mass spectrometry (LC-MS/MS) (Thermo Fisher Scientific, Waltham, MA, USA) [11].

Given that the whole data collected included a mixed population with varying BMIs and IR levels, we conducted subset analyses using BMI-matched and combined BMI- and IR-matched data extracted from the complete dataset.

Plasma CVRPs were measured by the slow off-rate modified aptamer (SOMA) scan platform [15]. Calibration was based on the standards previously described [16].

The slow off-rate modified aptamer (SOMAmer)-based protein array was utilized for protein quantification, following the previously outlined procedure [17,18]. Briefly, the following steps were performed with EDTA plasma samples: (1) the equilibration of SOMAmers for the binding of analyte and primer beads involved coupling the biotin moiety to a fully synthetic fluorophore-labeled SOMAmer through a photocleavable linker; (2) immobilization of the analyte/SOMAmers complex was carried out on streptavidin-substituted support; (3) using long-wave ultraviolet light, the analyte-SOMAmer complexes were cleaved and released into the solution; (4) analyte-SOMAmer complexes were immobilized on streptavidin support through analyte-borne biotinylation; (5) the elution of analyte-SOMAmer complexes was carried out, utilizing the released SOMAmers as surrogates for analyte quantification; and (6) quantification was performed through hybridization to SOMAmer complementary oligonucleotides. Normalization of raw intensities, hybridization, median signal, and calibration signal were standardized for each sample [15,16].

The SomaScan assay data standardization process involves several key steps to ensure data quality and comparability. First, hybridization normalization adjusts for well-to-well variations using hybridization control sequences. Next, intra-plate signal normalization is applied to calibrator and buffer replicates to correct for plate-specific biases. The process then includes plate scale standardization and calibration using a global calibrator reference to minimize between-plate variability. Quality control is performed by normalizing QC replicate signals against a global reference and checking the median QC replicate values against a global QC standard. Finally, individual sample signals are normalized using a global signal normalization reference to ensure consistency across all measurements. The average coefficient of variation (CV) is 6.1% [16].

Version 3.1 of the SOMAscan assay was used, targeting the 54 CVRPs, which are listed in Supplementary Table S1.

### 2.2. Data Analysis, Functional Enrichment, and Protein–Protein Interaction Network Analysis

SOMAscan proteomic data were quantile normalized and log-transformed for further statistical assessments. We used the linear models for microarray analysis (limma) for two class comparisons for detecting the CVRPs that were significantly regulated in the PCOS cohort. Any CVRP with a fold change of 1 and raw *p*-value < 0.05 was considered significant [19]. Supervised learning methods using univariate and multivariate stepwise logistic regression were performed to model the association of CVRPs with PCOS in these obese subjects. The significant CVRPs in the regression analysis were further assessed for their diagnostic accuracy by computing the Youden Index (YI) and then using the ROC (receiver operating curve) method. All tests were two tailed and *p* < 0.05 was considered significant. The statistical analysis was performed using R Bioconductor packages (RStudio 2023.06.2 Bioconductor(BiocManagerv1.30.25) and SPSS v 26.0.

The differentially expressed gene (DEG) list in PCOS participants was subjected to gene ontology (GO) analysis using the Database for Annotation, Visualization, and Integrated Discovery (DAVID) [https://david.ncifcrf.gov/, accessed on 18 August 2024]. Pathway enrichment using the KEGG database was also performed on the DAVID tool. FDR correction using the Benjamini–Hochberg technique was applied and an enriched term with an adjusted *p* value < 0.05 was considered significant.

As a part of further downstream analysis, the PCOS dysregulated CVRPs were submitted to STRING 12.0 database (https://string-db.org/; accessed on 10 October 2024) analysis for assessing the protein–protein interaction network (PPIN).

## 3. Results

### 3.1. Clinical Demographics

The baseline demographic data of the whole set of 97 non-PCOS and 147 PCOS participants are presented in Table 1. The PCOS subjects had significantly higher BMIs (*p* < 0.001) and elevated anti-Mullerian hormone (AMH) (*p* < 0.001), testosterone (*p* < 0.001) and free androgen index (FAI) (*p* < 0.001) levels. In addition, C-reactive protein (CRP) (*p* < 0.001), homeostasis model assessment–insulin resistance (HOMA-IR) (*p* < 0.05), and fasting blood glucose (*p* < 0.01) were elevated whilst PCOS women had a lower SHBG (*p* < 0.001). The participants in both the PCOS and control groups were age matched.

Subset analysis: BMI-matched (overweight/obese, BMI ≥ 26 kg/m^2^) and insulin resistance (IR)-(HOMA-IR < 1.9) plus BMI-matched subsets were also subjected to downstream analysis. Data subset analysis indicated that the frequency of overweight/obese patients having PCOS when HOMA-IR ≥ 1.9 is significantly higher compared to overweight/obese women with HOMA-IR < 1.9 (72.7% vs. 27.3%, Chi-square *p* value = 0.03).

**Figure 1:**
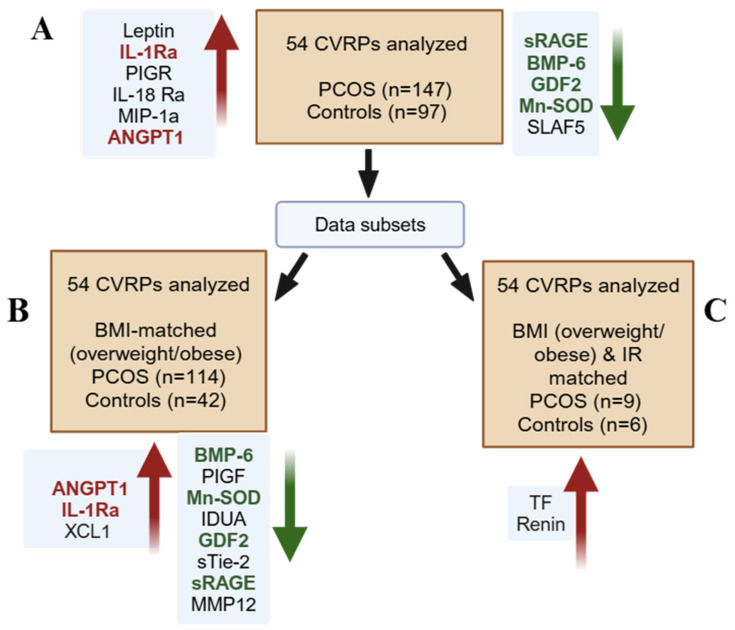
Analyses performed on whole set (**A**) and each subset (**B**,**C**) of women with and without polycystic ovary syndrome (PCOS). Overall cohort PCOS (n = 147) and controls (n = 97) in whom 54 cardiovascular risk proteins (CVRPs) were measured. Whole cohort was then divided into subsets: (**B**) body mass index (BMI) matched for BMI (≥26 kg/m^2^), PCOS (n = 114) and controls (n = 42); (**C**) matched for normal insulin resistance (HOMA-IR < 1.9) and BMI ≥ 26 kg/m^2^, PCOS (n = 9) and controls (n = 6). Significantly increased proteins shown with upward facing red arrows, significantly decreased proteins shown with downward facing green arrows. Cardiovascular risk proteins (CVRPs); bone morphogenetic protein 6 (BMP6); growth/differentiation factor 2 (GDF2); polymeric immunoglobulin receptor (PIGR); superoxide dismutase [Mn] mitochondrial (MnSOD); interleukin-18 receptor (IL-18Ra); C-C motif chemokine 3 (MIP-1a); SLAM family member 5 (SLAF5); angiopoietin-1 (ANGPT1); interleukin-1 receptor antagonist protein (IL-1Ra); advanced glycosylation end product-specific receptor, soluble (sRAGE); placenta growth factor (PIGF); lymphotactin (XCL1); alpha-L-iduronidase (IDUA); angiopoietin-1 receptor, soluble (s Tie-2); macrophage metalloelastase (MMP12); tissue factor (TF).

A summary of the division of the whole set into subsets is outlined in Figure 1.

### 3.2. Whole Set and Subset Analysis

A. Whole set: CVRPs that differed between PCOS (n = 147) and non-PCOS (n = 97) women in entire cohort.

**Figure 2:**
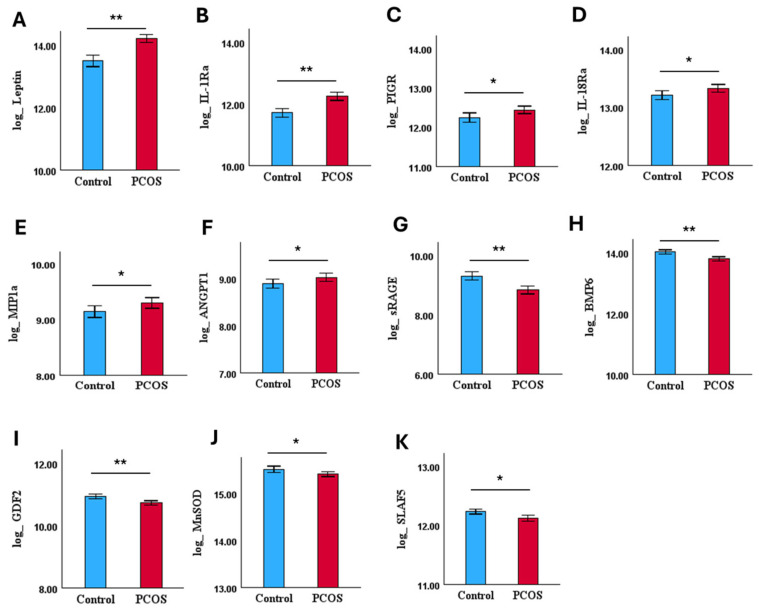
Bar plots of individual dysregulated CVRPs (mean ± SE) in whole cohort, control (n = 97) and PCOS (n = 147); (**A**–**F**) indicates levels of upregulated and (**G**–**K**) indicates levels of downregulated CVRPs in PCOS. ** *p* < 0.01, * *p* < 0.05.

Eleven of the 54 CVRPs were dysregulated in PCOS compared to non-PCOS women (Figure 2): leptin, interleukin-1 receptor antagonist protein (IL-1Ra), polymeric immunoglobulin receptor (PIGR), interleukin-18 receptor (IL-18Ra), C-C motif chemokine 3 (MIP-1a), and angiopoietin-1 (ANGPT1) were upregulated whilst advanced glycosylation end product-specific receptor, soluble (sRAGE), bone morphogenetic protein 6 (BMP6), growth/differentiation factor 2 (GDF2), superoxide dismutase [Mn] mitochondrial (MnSOD), and SLAM family member 5 (SLAF5) were downregulated relative to the controls (Figure 2, Table 2A).

B. Subset BMI-matched: CVRPs in BMI-matched (overweight/obese, BMI ≥ 26 kg/m^2^) PCOS (n = 114) and non-PCOS (n = 42) women.

**Figure 3:**
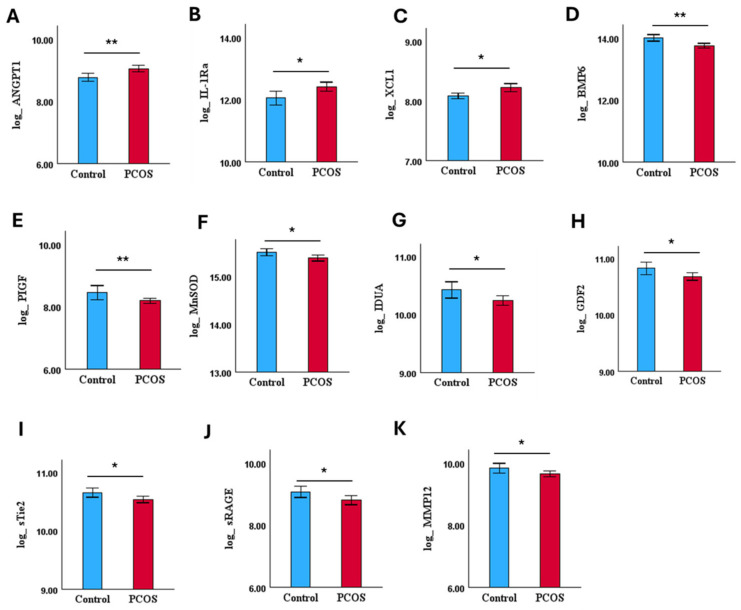
Bar plots of individual dysregulated CVRPs (mean ± SE) for BMI (≥26 kg/m^2^)-matched cohort, control (n = 47) and PCOS (n = 114); (**A**–**C**) indicates levels of upregulated and (**D**–**K**) indicates levels of downregulated CVRPs in PCOS. ** *p* < 0.01, * *p* < 0.05.

Again, 11 of the 54 CVRPs were dysregulated in overweight/obese PCOS compared to controls (Figure 3). Six of these CVRPs were common with the whole set: ANGPT1 and IL-1Ra were upregulated, and sRAGE, BMP6, GDF2, and Mn-SOD were downregulated. In addition, lymphotactin (XCL1) was upregulated and placenta growth factor (PIGF), alpha-L-iduronidase (IDUA), angiopoietin-1 receptor, soluble (sTie-2), and macrophage metalloelastase (MMP12) were downregulated (Figure 3, Table 2B).

C. Subset normal IR- and BMI-matched: CVRPs in BMI-matched (overweight/obese BMI ≥ 26 kg/m^2^) and normal IR-matched (HOMA-IR < 1.9) PCOS (n = 9) and non-PCOS (n = 6) women.

**Figure 4:**
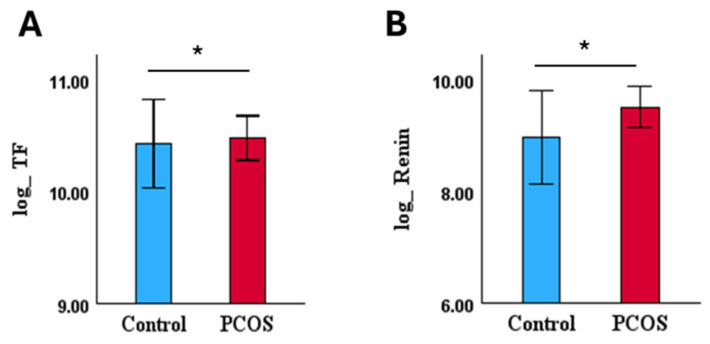
Bar plots of individual dysregulated CVRPs (mean ± SE) for matched normal insulin resistance (HOMA-IR < 1.9) and BMI ≥ 26 kg/m^2^ (**A**,**B**), PCOS (n = 9) and controls (n = 6), indicating upregulated CVRPs in PCOS, * *p* < 0.05.

In 2 out of 54 CVRPs, tissue factor (TF) and renin were upregulated in PCOS in this subset (Figure 4, Table 2C).

### 3.3. Multivariable Regression Analysis

The dysregulated proteins in the whole set were subjected to stepwise multivariable logistic regression to model their association with PCOS. The model had a Nagelkerke R Square of 0.31 and the variables included were BMP-6, IL-1Ra, ANGPT1, sRAGE, and leptin. Higher BMP-6 and sRAGE were noted in the non-PCOS versus the PCOS group and hence a negative regression parameter was associated with PCOS (BMP-6: B = −1.0, *p* = 0.03 and sRAGE: B = −0.60, *p* = 0.003). As per the models, the higher odds of having PCOS among women were associated with higher levels of ANGPT1 (OR 1.79, 95% CI: 0.93–3.43; *p* = 0.07), IL-1Ra (OR 1.64, 95% CI 1.03–2.62, *p* = 0.03) and leptin (OR 1.84, 95% CI 1.23–2.77, *p* = 0.003).

The dysregulated proteins in the BMI-matched (overweight/obese, BMI ≥ 26 kg/m^2^) individuals were subjected to multivariable logistic regression to model their association with PCOS. The model had a Nagelkerke R Square of 0.365. The model indicated that participants who were overweight/obese with PCOS were more likely to have higher levels of ANGPT1 (OR 3.85, 95% CI: 1.05–13.35, *p* < 0.001), programmed cell death 1 ligand 2 (PD-L2) (OR 2.22, 95% CI: 0.78–8.07, *p* < 0.004), and IL1-Ra (OR 0.98, 95% CI 0.31–8.35, *p* = 0.004). Negative regression terms were associated with PIGF, sRAGE, and Dickkopf-related protein 1 (DKK1).

The IR-matched (HOMA-IR < 1.9) plus BMI-matched (overweight/obese, BMI ≥ 26 kg/m^2^) data subset failed to develop any supervised learning models.

### 3.4. ROC Curve Analysis

**Figure 5:**
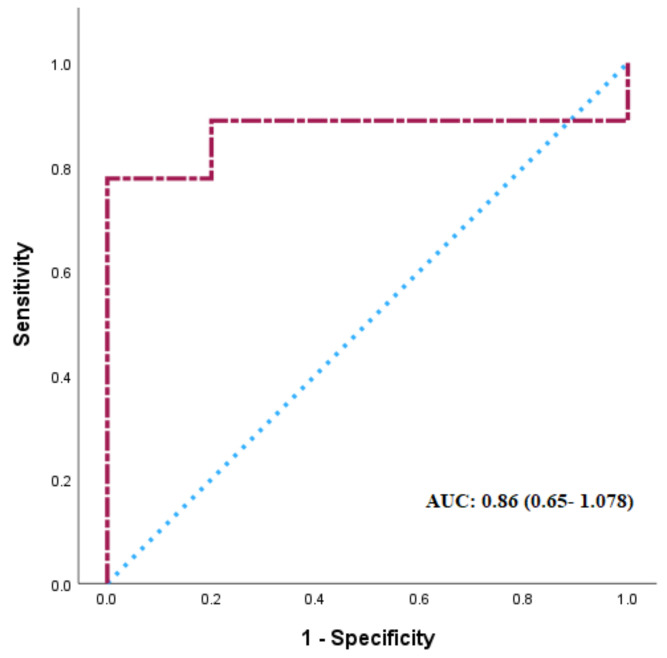
The ROC curve of renin. The area under the curve (AUC) indicates the potential of renin in discriminating women with PCOS from the controls in the subset of women with normal insulin resistance (HOMA-IR < 1.9) and BMI in the overweight/obese range (BMI ≥ 26 kg/m^2^).

ROC curve analysis was performed with the IR-matched (HOMA-IR < 1.9) plus BMI-matched (overweight/obese, BMI ≥ 26 kg/m^2^) data subset to identify the CVRPs that could delineate PCOS in this subpopulation. The analysis showed that among the 54 CVRPs, renin was able to distinguish PCOS in this subset. The area under the curve (AUC) for renin was 0.86 (95% CI 0.65–1.078, *p* = 0.001). According to the ROC curves and Youden’s Index, the optimal cutoff value of renin expression level was 596.3 RFU, with 77.8% sensitivity and 99.9% specificity (Figure 5).

### 3.5. Protein–Protein Interaction

**Figure 6:**
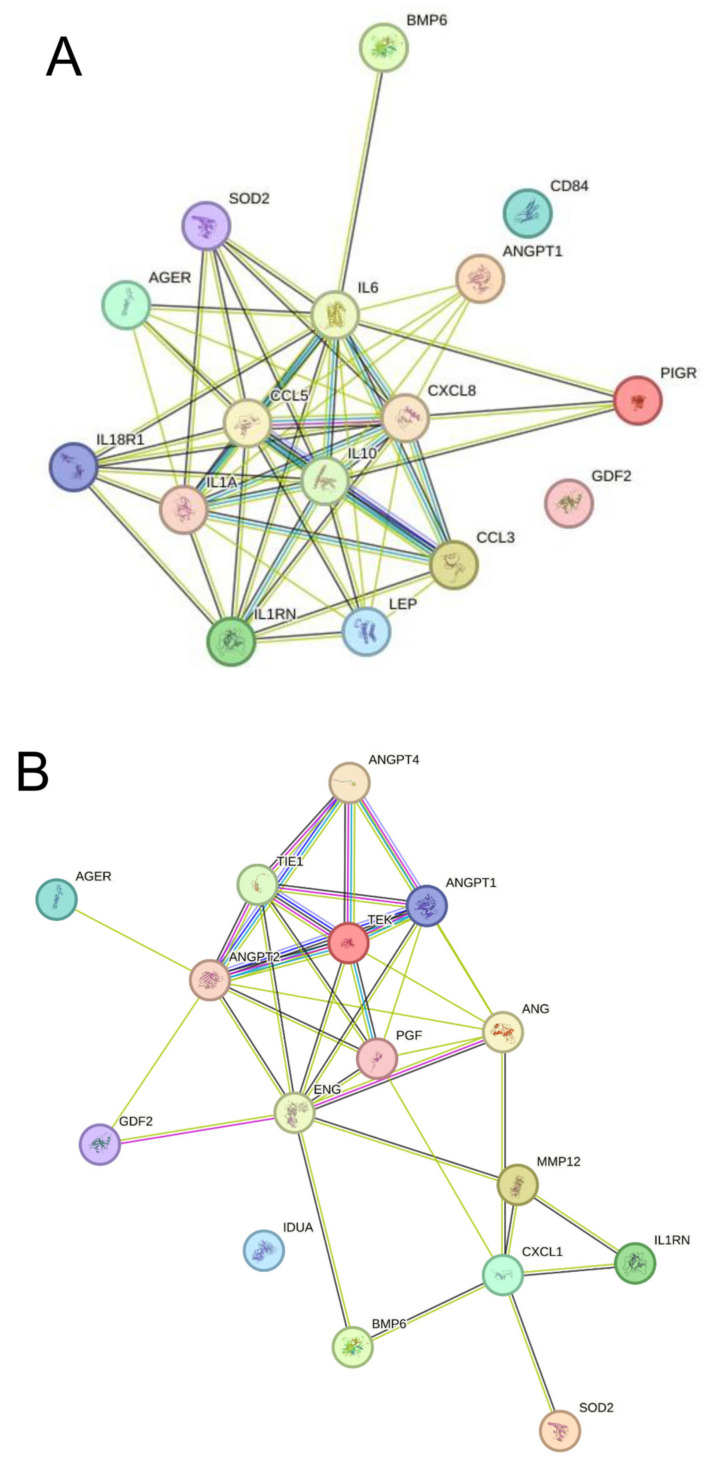
STRING (version 12.0) protein–protein interaction network between cardiovascular risk biomarkers (CVRPs) that differed (**A**) between whole set of women with and without PCOS and their predicted immediate binding partners and (**B**) in subset of matched overweight/obese women (BMI ≥ 26 kg/m^2^) with and without PCOS. ‘Co-expression’ is indicated by black edge. Interactions obtained through text mining indicated by yellow edges.

STRING 12.0 (Search Tool for the Retrieval of Interacting Genes) was used to visualize the known and predicted protein–protein interaction for proteins that were upregulated in the following populations: CVRPs in the whole set of participants (PCOS vs. non-PCOS) (Figure 6A) and CVRPs in BMI-matched (overweight/obese, BMI ≥ 26 kg/m^2^) PCOS vs. non-PCOS (Figure 6B) (https://string-db.org/; accessed on 10 October 2024) groups. The figures represent interactions between the upregulated CVRPs and their immediate interacting partners.

### 3.6. Functional Enrichment Analysis for Dysregulated Proteins

A comprehensive analysis identified eleven dysregulated cardiovascular risk proteins (CVRPs) in the whole set of participants (PCOS vs. non-PCOS) (Table 3A) and eleven dysregulated CVRPs in the BMI-matched subset (overweight/obese, BMI ≥ 26 kg/m^2^) PCOS vs. non-PCOS (Table 3B) groups. Further investigation through functional enrichment and gene ontology (GO) analysis using the DAVID tool highlighted an increase in GO terms linked to the regulation of cytokines and inflammatory responses, critical pathways known to be actively dysregulated in PCOS and cardiovascular disease. These findings suggest a potential pivotal role for these proteins and pathways in the shared pathogenesis of PCOS and cardiovascular disease.

## 4. Discussion

This research provides insights into the dysregulation of CVRPs in women with PCOS, especially in those who are overweight/obese. The dysregulated proteins reported here emphasize the intricate, though complex, interplay between metabolic and cardiovascular pathways in PCOS, implicating these proteins as contributors to the increased risk of CVD in these women.

### 4.1. Dysregulation in CVRPs in PCOS Women (Whole Set)

In this exploratory investigation, 11 CVRPs were dysregulated in the entire PCOS group where IR and BMI were unmatched: leptin, IL-1Ra, PIGR, IL-18Ra, MIP-1a, and ANGPT1 were upregulated whereas sRAGE, BMP6, GDF2, Mn-SOD, and SLAF 5 were downregulated.

Leptin, an adipocyte-derived pro-inflammatory adipokine, contributes to the low-grade inflammatory state in overweight/obese individuals and is implicated in CVD events, with hyperleptinemia linked to coronary heart disease and heart failure [20]. Beyond its cardiovascular effects, leptin plays a crucial role in reproductive processes [21], highlighting its diverse impact on multiple physiological functions in the body. Elevated serum leptin levels were reported in overweight/obese women with PCOS, and are linked to hyperandrogenemia and IR, key features of the syndrome [22,23]. Leptin exerts significant peripheral effects that may contribute to the development of cardiometabolic disorders by promoting vascular inflammation, increasing oxidative stress, and inducing hypertrophy of vascular smooth muscle cells [24].

Interleukin 1 receptor antagonist (IL-1Ra) is a critical mediator of inflammatory processes that binds to the IL-1 receptor, blocking IL-1 alpha and beta without inducing signaling; IL-1Ra was found to be upregulated in our study, and is also elevated in nonobese PCOS patients [10]. Studies have shown that IL-1Ra gene polymorphisms, particularly allele II in intron 2, are strongly associated with metabolic features of PCOS [25], and elevated IL-1Ra levels may predict impaired glucose metabolism regardless of BMI [26]. IL-1Ra plays a significant role in the pathophysiology of PCOS and may contribute to CVD risk in these patients [10]. Elevated levels of IL-1Ra in women with PCOS correlate with IR, obesity, and impaired glucose metabolism [26].

Polymeric immunoglobulin receptor (PIGR) was upregulated in PCOS. PIGR is expressed in the intestine, bronchus, salivary glands, renal tubule, and uterus [27]. PIGR is essential in mucosal immunity for transporting dimeric IgA (dIgA) across epithelial cells. However, its role in PCOS is unexplored.

IL-18Ra, a pro-inflammatory cytokine, was elevated in women with PCOS. Elevated levels of IL-18 and its receptor were reported in women with PCOS, correlating with IR, obesity, and hyperandrogenism [28] and it is implicated in the inflammatory processes that contribute to metabolic syndrome, a condition associated with an increased risk of cardiovascular events [29].

An increase in MIP-1a was also observed in PCOS, in accordance with prior reports [30,31]. Elevated levels of MIP-1a in PCOS activate the phosphatidylinositol 3-kinase/protein kinase B (PI3K/AKT) and mitogen-activated protein kinase (MAPK) signaling pathways, leading to the increased production of pro-inflammatory cytokines and enhanced inflammatory responses, potentially contributing to cardiovascular risk [32,33].

ANGPT1 plays a significant role in the pathophysiology of PCOS and its associated cardiovascular risk [34]. Additionally, treatment with ANGPT1 reduces the risk of diet-induced obesity [35]. Our study found an increase in ANGPT1 expression in the PCOS overweight/obese cohort versus their non-PCOS counterparts, that agrees with other reports of elevated levels in PCOS, suggesting a compensatory mechanism in response to the heightened vascular permeability driven by other factors like vascular endothelial growth factor (VEGF) [36].

sRAGE was decreased in women with PCOS in this study. sRAGE has an inverse relationship with AGEs and may serve as a protective factor against cardiovascular complications in PCOS [37]. Decreased sRAGE levels in women with PCOS may exacerbate the harmful effects of AGEs, potentially contributing to long-term metabolic and cardiovascular risks mediated through chronic inflammation and IR [38,39,40]. sRAGE may serve as a protective factor against the cardiovascular complications associated with PCOS by binding to AGEs and thus mitigating their harmful effects on vascular health [37].

Bone morphogenetic protein 6 (BMP6) was found to be decreased here in the PCOS subjects though, in a study of circulating BMP6 levels using a less sensitive detection method, BMP6 was not found to be detectable [41]. BMP6 is involved in regulating ovarian function, particularly in follicle development and oocyte maturation, by modulating intercellular communication within the ovary. Dysregulation of BMP6 signaling was linked to the pathogenesis of PCOS, contributing to ovulatory dysfunction [42,43] and associated metabolic disturbances, which can elevate cardiovascular risk in affected women.

Growth/differentiation factor 2 (GDF2), also known as bone morphogenetic protein 9 (BMP9), was found to be reduced in the PCOS versus control groups. GDF2 is involved in the regulation and control of ovarian folliculogenesis [44]. Circulating BMP9 levels were found to correlate negatively with cardiovascular risk factors, such as hypertension and coronary heart disease [45,46]. Lower levels of BMP9 are associated with an increased risk of these conditions, suggesting that BMP9 could serve as a potential biomarker for CVD progression in individuals, including those with metabolic disorders such as PCOS.

Superoxide dismutase [Mn] (MnSOD) was found to be decreased in women with PCOS. Studies on serum SOD activity in PCOS patients have reported conflicting results [47], with some studies suggesting elevated SOD levels in PCOS [48,49], whilst others suggest the opposite [50]. MnSOD plays a protective role by reducing superoxide levels in vascular tissues [51], protecting against CVD, as oxidative stress is a known contributor to cardiovascular pathology. In patients with PCOS, the risk of CVD is increased due to the associated IR and metabolic syndrome, and a reduction in MnSOD activity may be detrimental [49,52].

SLAF5, also known as CD84, was downregulated in women with PCOS in our study. SLAF5, a homophilic cell surface glycoprotein, is primarily expressed at peak levels on macrophages, dendritic cells, platelets and, to a lesser extent, on immune cells such as B lymphocytes. There is a paucity of information about the role of SLAF5 in PCOS. CD84 is shown to be highly expressed in patients with chronic kidney disease (KD) with coronary arteritis [53]. CD84 likely plays an important role in the pathogenesis of chronic inflammation, but it is unclear whether it plays a protective or a deleterious role.

### 4.2. Dysregulation of CVRPs in BMI-Matched PCOS Subset

When only BMI-matched (overweight/obese, BMI ≥ 26 kg/m^2^) candidates were considered, again 11 of the 54 CVRPs were dysregulated in obese/overweight PCOS individuals compared to their non-PCOS counterparts. Among these, ANGPT1, IL-1Ra, and XCL1 were upregulated whereas BMP6, PIGF, Mn-SOD, IDUA, GDF2, sTie-2, sRAGE, and MMP12 were downregulated.

XCL1, also known as lymphotactin, was upregulated in PCOS individuals in this subset, and is a C-class chemokine produced by T cells and natural killer cells in response to inflammatory and infectious stimuli. It predominantly exerts its effects by binding to and activating the XCR1 receptor [54]. There are no reports to date about this protein in the context of PCOS.

ANGPT1 and IL-1Ra were again upregulated in the BMI-matched PCOS cohort and their roles in the pathophysiology of PCOS are noted above.

Decreased levels of PIGF were found in BMI-matched PCOS women. Chen et al. investigated placental growth factor (PIGF), a protein that stimulates the growth and survival of endothelial cells under ischemic conditions, and showed that a high ratio of circulating PIGF to the cell stress marker TRAIL receptor-2 indicates a lower cardiovascular risk, indicative of the plausible protective action of PIGF in CVDs [55].

IDUA was downregulated in the BMI-matched PCOS cohort. IDUA (α-L-iduronidase) is involved in the breakdown of glycosaminoglycans (GAGs) and its deficiency may lead to an accumulation of GAGs thereby negatively impacting cardiovascular health [56]. There is no direct evidence of IDUA expression in relation to obesity and PCOS.

Our study reports the downregulation of serum s-Tie2 in overweight/obese BMI-matched PCOS women. The soluble form of the Tie2 receptor (s-Tie2) binds to angiopoietins and is essential for vascular stability and remodeling. Its direct role in obesity and PCOS have not previously been reported [57] though Scotti et al. reported no difference in sTie2 from follicular fluid in PCOS versus control individuals [58].

MMP12 was downregulated in overweight/obese BMI-matched PCOS women. MMP12 degrades elastin and promotes macrophage recruitment, increasing the risk for CVDs [59]. MMP12 expression is associated with metabolic dysfunction and, in contrast to our findings here, was reported to be elevated in obesity, which contributes to alterations in the extracellular matrix (ECM) [60]. PCOS women are at an increased risk of developing preeclampsia, a condition that shares common cardiovascular markers, such as MMP12. This was demonstrated for the angiogenic marker CD93, which has a pathogenic role both in the context of obesity and cardiovascular disease [61], as well as in preeclampsia, emphasizing the relevance of these markers in broader pathological contexts.

sRAGE, BMP6, GDF2, and Mn-SOD were downregulated in overweight/obese BMI-matched PCOS women. These CVRPs play a protective role against CVDs and their downregulation in PCOS women is indicative of increased cardiovascular risk.

### 4.3. Dysregulation of CVRPs in IR-Matched Plus BMI-Matched PCOS Subset

When both BMI (overweight/obese, BMI ≥ 26 kg/m^2^) and IR (HOMA-IR < 1.9) were accounted for, only two proteins, tissue factor (TF) and renin, were dysregulated (upregulated), indicating that both TF and renin were independent of both obesity and insulin resistance, suggesting that these may be CVRPs inherent to PCOS. TF, a transmembrane glycoprotein, serves as the primary initiator of blood coagulation and is induced on monocytes and endothelial cells by inflammatory stimuli such as endotoxin, tumor necrosis factor and IL-113 [62]. Elevated TF levels are associated with increased cardiovascular risk, acute coronary syndrome, and PCOS. In accordance with our results, the increased expression of TF in PCOS is independent of obesity [63].

In the BMI- and IR-matched subjects, renin was upregulated in PCOS women. Renin is the first limiting step in the Renin Angiotenin Aldosterone System (RAAS) and is also a biomarker for CVD [64]. Renin plays a significant role in the metabolic abnormalities observed in polycystic ovary syndrome (PCOS), particularly in relation to IR, as women with PCOS exhibit higher renin levels that positively correlate with insulin concentrations and HOMA-IR [65,66]. This suggests a complex interplay between RAAS and insulin signaling pathways [67,68].

Functional enrichment analysis of the dysregulated proteins revealed significant pathways linked to cytokine production regulation, endothelial cell proliferation, and inflammatory responses. This agrees with the chronic inflammation and vascular dysfunction inherent to PCOS [69] whilst providing a link between PCOS and CVDs [12].

Six dysregulated proteins were common between the whole PCOS cohort and the BMI-matched PCOS cohort, of which ANGPT1 and IL-1Ra were upregulated whereas sRAGE, BMP6, GDF2, and Mn-SOD were downregulated. Thus, CVRPs may serve as potential biomarkers for cardiovascular risk in overweight/obese women with PCOS. Of particular interest is the role of ANGPT1 and leptin, which are associated with inflammation and vascular function. Four CVRPs were positively associated with obesity irrespective of age, leptin [70], IL-1Ra [71], IL-18 Ra [72], and MIP-1a [73], and all four were upregulated in PCOS. Conversely, sRAGE [74], Mn-SOD [51,75] BMP6, and GDF2 [76] were downregulated in the overweight/obese PCOS cohort and all of these were reported to have reduced expression in obesity with metabolic syndrome; it is therefore not surprising that the levels of these proteins were reduced in the overweight/ obese subset of women, both with and without PCOS, though it appears that PCOS caused further downregulation.

A multivariate regression analysis model was used here to investigate the link between specific CVRPs and PCOS. The analysis indicates that elevated levels of ANGPT1, IL-1Ra, and leptin are associated with a higher risk for PCOS. These proteins have crucial roles in pathways related to inflammation and metabolic dysfunction in PCOS. Conversely, a negative regression parameter was associated with BMP6 and sRAGE in PCOS, indicating a compromised regulatory mechanism in PCOS. Renin was found to distinguish PCOS in the BMI- and IR-matched women, as seen in the ROC curve analysis, suggesting its value as a biomarker in this particular subset.

STRING analysis of the 11 dysregulated proteins from the whole PCOS cohort and the BMI-matched PCOS cohort indicate that, although these proteins have limited direct interactions, they are well connected through their immediate binding partners, such as interleukin 10 (IL10), C-C motif chemokine ligand 3 (CCL3) and interleukin 1 alpha (IL1A), which are reported as active members in cytokine regulation, cytokine–cytokine interaction, inflammatory responses and, for example, angiopoietin 2 (ANGPT2), angiopoietin 4 (ANGPT4), and angiogenin (ANG) that have specific roles in vascular function. Thus, the dysregulated proteins identified here do not act in isolation but rather as part of a broader network influencing metabolic and cardiovascular health. The co-expression and interaction patterns suggest that targeting these protein pathways could be a viable strategy for mitigating cardiovascular risk associated with PCOS.

The results in this study differed to the CVRPs that were reported in a nonobese PCOS study [10], likely due to the influence of the increased weight that was associated with the CVRPs reported here and that would have not been a factor in that study. In addition, the women in this study were all PCOS phenotype A and it is unclear what the phenotype was in the nonobese study, though these patients tend to be phenotype B or C, and C is less frequently associated with an increased cardiovascular risk [77]. In view of the potential confounding effects of over-the-counter medication (such as anti-inflammatory agents and herbal preparations), these were specifically excluded in the population studied to ensure that the protein changes reported were not pharmacologically exaggerated or suppressed.

The limitations of this study include its small sample size and the fact that it was conducted solely on a Caucasian population, which may restrict the generalizability of the findings. To confirm these results, similar studies should be conducted in diverse ethnic groups. Additionally, further molecular-level analyses are necessary to establish the potential role of predictive protein candidates, such as IL1-Ra and leptin, as clinical indicators of PCOS in overweight individuals. In addition, further studies on CV risk in PCOS should also account for the PCOS phenotype.

## 5. Conclusions

In conclusion, a combination of upregulated obesity-related CVRPs (ANGPT1, IL, 1Ra, XCL1) and downregulated cardioprotective proteins (sRAGE, BMP6, Mn-SOD, GDF2) in PCOS may contribute to the increased risk of CVDs in overweight women with PCOS. The observed upregulation of TF and renin in the BMI- and IR-matched PCOS subgroup, despite the limited sample size, suggests a potential association with cardiovascular risks in these patients, warranting further investigation in larger cohorts.

## Figures and Tables

**Table 1:** Demographics and baseline hormonal and metabolic parameters in women with PCOS (n = 147) and non-PCOS (n = 97) (mean ± SD). BMI—body mass index; HOMA-IR—homeostasis model assessment–insulin resistance; CRP—C reactive protein; SHBG—sex hormone binding globulin; AMH—anti-Müllerian hormone.

	PCOS (n = 147)	Non-PCOS (n = 97)	*p* Value
Age (years)	27.7 ± 6.3	29.3 ± 6.6	0.06
BMI (kg/m^2^)	34.0 ± 7.6	26.7 ± 6.5	<0.001
Insulin uIU/mL	6.3 ± 3.2	10.6 ± 6.4	0.002
AMH (ng/mL)	40.8 ± 23.4	20.1 ± 18.1	<0.001
Testosterone (nmol/L)	1.6 ± 1.0	1.1 ± 0.5	<0.001
SHBG (nmol/L)	41.2 ± 38.6	74.2 ± 74.4	<0.001
Free androgen index (FAI)	6.3 ± 6.1	2.3 ± 1.6	<0.001
Fasting glucose (nmol/L)	4.9 ± 1.0	4.7 ± 0.7	0.005
HOMA-IR	2.2 ± 1.6	1.3 ± 0.7	0.010
CRP (mg L^−1^)	4.7 ± 4.8	2.3 ± 3.8	<0.001

**Table 2:** (**A**) Whole set: CVRPs that differed between PCOS (n = 147) and non-PCOS (n = 97) women in entire cohort. (**B**) Subset BMI-matched: CVRPs in BMI-matched (overweight/obese, BMI ≥ 26 kg/m^2^) PCOS (n = 114) and non-PCOS (n = 42) women. (**C**) Subset normal IR- and BMI-matched: CVRPs in BMI-matched (overweight/obese BMI ≥ 26 kg/m^2^) and normal IR-matched (HOMA-IR < 1.9) PCOS (n = 9) and non-PCOS (n = 6) women.

**(A)**				
**Gene**	**logFC**	**Average Expression**	**t**	***p*** **Value**
*Leptin*	0.71	13.9	6.56	<0.001
*IL-1Ra*	0.53	12.03	5.33	<0.001
*sRAGE*	−0.46	9.01	−4.67	<0.001
*BMP-6*	−0.23	13.88	−4.50	<0.001
*GDF2*	−0.21	10.81	−4.00	<0.001
*PIGR*	0.19	12.36	2.53	0.01
*Mn-SOD*	−0.10	15.45	−2.50	0.01
*IL-18 Ra*	0.11	13.28	2.24	0.02
*MIP-1a*	0.15	9.23	2.15	0.03
*SLAF5*	−0.06	12.19	−2.13	0.03
*ANGPT1*	0.13	8.97	1.94	0.04
**(B)**				
**Gene**	**logFC**	**Average Expression**	**t**	***p*** **Value**
*BMP6*	−0.25	13.82	−3.56	<0.001
*ANGPT1*	0.28	8.98	2.97	<0.001
*PIGF*	−0.26	8.26	−2.73	0.007
*IL1Ra*	0.37	12.32	2.69	0.007
*XCL1*	0.13	8.18	2.33	0.02
*Mn-SOD*	−0.12	15.41	−2.26	0.02
*IDUA*	−0.17	10.29	−2.24	0.02
*GDF2*	−0.14	10.72	−2.20	0.03
*sTie-2*	−0.11	10.57	−2.17	0.03
*sRAGE*	−0.27	8.88	−2.11	0.03
*MMP12*	−0.18	9.71	−2.07	0.04
**(C)**				
**Gene**	**logFC**	**Average Expression**	**t**	***p*** **Value**
*TF*	0.37	10.63	2.559517	0.02
*Renin*	0.54	9.143917	2.101566	0.04

Cardiovascular risk proteins (CVRPs); bone morphogenetic protein 6 (BMP6); growth/differentiation factor 2 (GDF2); polymeric immunoglobulin receptor (PIGR); superoxide dismutase [Mn] mitochondrial (MnSOD); interleukin-18 receptor (IL-18Ra); C-C motif chemokine 3 (MIP-1a); SLAM family member 5 (SLAF5); angiopoietin-1 (ANGPT1); interleukin-1 receptor antagonist protein (IL-1Ra); advanced glycosylation end product-specific receptor, soluble (sRAGE); placenta growth factor (PIGF); lymphotactin (XCL1); alpha-L-iduronidase (IDUA); angiopoietin-1 receptor, soluble (s Tie-2); macrophage metalloelastase (MMP12); tissue factor (TF).

**Table 3:** Significant enrichment terms carried out using DAVID online tool for cardiovascular risk biomarkers in PCOS and non-PCOS women. (**A**) Whole set: Significant enrichment terms for CVRPs that differed between whole cohort of PCOS and non-PCOS women. (**B**) Subset BMI-matched: Significant enrichment terms for CVRPs that differed between BMI-matched overweight/obese subset of PCOS and non-PCOS women.

**(A)**			
**GO Terms**	**Count**	***p*-Value**	**Benjamini**
**Biological processes**			
regulation of cytokine production	7	1.7 × 10^−6^	6.9 × 10^−4^
positive regulation of tumor necrosis factor production	4	2.10 × 10^−5^	7.30 × 10^−3^
regulation of cell communication	9	5.60 × 10^−5^	3.20 × 10^−3^
response to stress	8	5.40 × 10^−4^	1.40 × 10^−2^
vascular process in circulatory system	5	8.40 × 10^−6^	1.30 × 10^−3^
**Pathway enrichment of dysregulated genes**			
KEGG PATHWAY: Cytokine–cytokine receptor interaction	6	4.70 × 10^−6^	1.70 × 10^−4^
**(** **B)**			
**GO Terms**	**Count**	***p*-Value**	***Benjamini***
**Biological processes**			
positive regulation of endothelial cell proliferation	4	5.5 × 10^−6^	1.4 × 10^−3^
positive regulation of protein phosphorylation	4	8.1 × 10^−5^	9.3 × 10^−3^
response to hypoxia	4	1.3 × 10^−4^	9.3 × 10^−3^
sprouting angiogenesis	3	1.4 × 10^−4^	9.3 × 10^−3^
**Cellular component**			
extracellular space	7	1.3 × 10^−4^	2.6 × 10^−3^
extracellular region	7	1.5 × 10^−4^	2.6 × 10^−3^
**Molecular function**			
growth factor activity	4	7.0 × 10^−5^	3.5 × 10^−3^

## Data Availability

The original contributions presented in this study are included in the article/Supplementary Materials. Further inquiries can be directed to the corresponding author.

## Supplementary Materials

The following supporting information can be downloaded at: https://www.mdpi.com/article/10.3390/biom15010004/s1, Table S1: Cardiovascular risk proteins that were analysed using SOMAscan.

## References

[1] Salari N., Nankali A., Ghanbari A., Jafarpour S., Ghasemi H., Dokaneheifard S., Mohammadi M. (2024). Global prevalence of polycystic ovary syndrome in women worldwide: A comprehensive systematic review and meta-analysis. Arch. Gynecol. Obstet..

[2] Diamanti-Kandarakis E., Kouli C.R., Bergiele A.T., Filandra F.A., Tsianateli T.C., Spina G.G., Zapanti E.D., Bartzis M.I. (1999). A survey of the polycystic ovary syndrome in the Greek island of Lesbos: Hormonal and metabolic profile. J. Clin. Endocrinol. Metab..

[3] Sathyapalan T., Atkin S.L. (2012). Recent advances in cardiovascular aspects of polycystic ovary syndrome. Eur. J. Endocrinol..

[4] Che Y., Yu J., Li Y.S., Zhu Y.C., Tao T. (2023). Polycystic Ovary Syndrome: Challenges and Possible Solutions. J. Clin. Med..

[5] Siddiqui S., Mateen S., Ahmad R., Moin S. (2022). A brief insight into the etiology, genetics, and immunology of polycystic ovarian syndrome (PCOS). J. Assist. Reprod. Genet..

[6] Cena H., Chiovato L., Nappi R.E. (2020). Obesity, Polycystic Ovary Syndrome, and Infertility: A New Avenue for GLP-1 Receptor Agonists. J. Clin. Endocrinol. Metab..

[7] Ollila M.M., Arffman R.K., Korhonen E., Morin-Papunen L., Franks S., Junttila J., Piltonen T.T. (2023). Women with PCOS have an increased risk for cardiovascular disease regardless of diagnostic criteria-a prospective population-based cohort study. Eur. J. Endocrinol..

[8] Powell-Wiley T.M., Poirier P., Burke L.E., Despres J.P., Gordon-Larsen P., Lavie C.J., Lear S.A., Ndumele C.E., Neeland I.J., Sanders P. (2021). Obesity and Cardiovascular Disease: A Scientific Statement From the American Heart Association. Circulation.

[9] Osibogun O., Ogunmoroti O., Michos E.D. (2020). Polycystic ovary syndrome and cardiometabolic risk: Opportunities for cardiovascular disease prevention. Trends Cardiovasc. Med..

[10] Nandakumar M., Das P., Sathyapalan T., Butler A.E., Atkin S.L. (2024). A Cross-Sectional Exploratory Study of Cardiovascular Risk Biomarkers in Non-Obese Women with and without Polycystic Ovary Syndrome: Association with Vitamin D. Int. J. Mol. Sci..

[11] Cunningham T.K., Allgar V., Dargham S.R., Kilpatrick E., Sathyapalan T., Maguiness S., Mokhtar Rudin H.R., Abdul Ghani N.M., Latiff A., Atkin S.L. (2019). Association of Vitamin D Metabolites With Embryo Development and Fertilization in Women With and Without PCOS Undergoing Subfertility Treatment. Front. Endocrinol..

[12] Rotterdam ESHRE/ASRM-Sponsored PCOS Consensus Workshop Group (2004). Revised 2003 consensus on diagnostic criteria and long-term health risks related to polycystic ovary syndrome (PCOS). Hum. Reprod..

[13] Legro R.S., Arslanian S.A., Ehrmann D.A., Hoeger K.M., Murad M.H., Pasquali R., Welt C.K. (2013). Diagnosis and Treatment of Polycystic Ovary Syndrome: An Endocrine Society Clinical Practice Guideline. J. Clin. Endocrinol. Metab..

[14] World Health Organization (2008). Waist Circumference and Waist-Hip Ratio: Report of a WHO Expert Consultation.

[15] Kahal H., Halama A., Aburima A., Bhagwat A.M., Butler A.E., Grauman J., Suhre K., Sathyapalan T., Atkin S.L. (2020). Effect of induced hypoglycemia on inflammation and oxidative stress in type 2 diabetes and control subjects. Sci. Rep..

[16] Kraemer S., Vaught J.D., Bock C., Gold L., Katilius E., Keeney T.R., Kim N., Saccomano N.A., Wilcox S.K., Zichi D. (2011). From SOMAmer-based biomarker discovery to diagnostic and clinical applications: A SOMAmer-based, streamlined multiplex proteomic assay. PLoS ONE.

[17] Gold L., Ayers D., Bertino J., Bock C., Bock A., Brody E.N., Carter J., Dalby A.B., Eaton B.E., Fitzwater T. (2010). Aptamer-based multiplexed proteomic technology for biomarker discovery. PLoS ONE.

[18] Suhre K., Arnold M., Bhagwat A.M., Cotton R.J., Engelke R., Raffler J., Sarwath H., Thareja G., Wahl A., DeLisle R.K. (2017). Connecting genetic risk to disease end points through the human blood plasma proteome. Nat. Commun..

[19] Berrone E., Chiorino G., Guana F., Benedetti V., Palmitessa C., Gallo M., Calvo A., Casale F., Manera U., Favole A. (2023). SOMAscan Proteomics Identifies Novel Plasma Proteins in Amyotrophic Lateral Sclerosis Patients. Int. J. Mol. Sci..

[20] Poetsch M.S., Strano A., Guan K. (2020). Role of Leptin in Cardiovascular Diseases. Front. Endocrinol..

[21] Perez-Perez A., Sanchez-Jimenez F., Maymo J., Duenas J.L., Varone C., Sanchez-Margalet V. (2015). Role of leptin in female reproduction. Clin. Chem. Lab. Med..

[22] Chakrabarti J. (2013). Serum leptin level in women with polycystic ovary syndrome: Correlation with adiposity, insulin, and circulating testosterone. Ann. Med. Health Sci. Res..

[23] Daghestani M.H., Daghestani M., Daghistani M., El-Mazny A., Bjorklund G., Chirumbolo S., Al Saggaf S.H., Warsy A. (2018). A study of ghrelin and leptin levels and their relationship to metabolic profiles in obese and lean Saudi women with polycystic ovary syndrome (PCOS). Lipids Health Dis..

[24] Koh K.K., Park S.M., Quon M.J. (2008). Leptin and cardiovascular disease: Response to therapeutic interventions. Circulation.

[25] Rashid N., Nigam A., Saxena P., Jain S.K., Wajid S. (2017). Association of IL-1beta, IL-1Ra and FABP1 gene polymorphisms with the metabolic features of polycystic ovary syndrome. Inflamm. Res..

[26] Luotola K., Piltonen T.T., Puurunen J., Tapanainen J.S. (2016). IL-1 receptor antagonist levels are associated with glucose tolerance in polycystic ovary syndrome. Clin. Endocrinol..

[27] Krajci P., Solberg R., Sandberg M., Oyen O., Jahnsen T., Brandtzaeg P. (1989). Molecular cloning of the human transmembrane secretory component (poly-Ig receptor) and its mRNA expression in human tissues. Biochem. Biophys. Res. Commun..

[28] Ahmad R., Thomas R., Kochumon S., Sindhu S. (2017). Increased adipose tissue expression of IL-18R and its ligand IL-18 associates with inflammation and insulin resistance in obesity. Immun. Inflamm. Dis..

[29] Dinarello C.A. (2006). Interleukin 1 and interleukin 18 as mediators of inflammation and the aging process. Am. J. Clin. Nutr..

[30] Vasyukova E., Zaikova E., Kalinina O., Gorelova I., Pyanova I., Bogatyreva E., Vasilieva E., Grineva E., Popova P. (2023). Inflammatory and Anti-Inflammatory Parameters in PCOS Patients Depending on Body Mass Index: A Case-Control Study. Biomedicines.

[31] Wu Z., Fang L., Li Y., Yan Y., Thakur A., Cheng J.C., Sun Y.P. (2021). Association of circulating monocyte chemoattractant protein-1 levels with polycystic ovary syndrome: A meta-analysis. Am. J. Reprod. Immunol..

[32] Lentzsch S., Gries M., Janz M., Bargou R., Dörken B., Mapara M.Y. (2003). Macrophage inflammatory protein 1-alpha (MIP-1alpha) triggers migration and signaling cascades mediating survival and proliferation in multiple myeloma (MM) cells. Blood.

[33] Zhang G., Liu H.B., Zhou L., Cui X.Q., Fan X.H. (2018). CCL3 participates in the development of rheumatoid arthritis by activating AKT. Eur. Rev. Med. Pharmacol. Sci..

[34] Tal R., Seifer D.B., Grazi R.V., Malter H.E. (2013). Angiopoietin-1 and angiopoietin-2 are altered in polycystic ovarian syndrome (PCOS) during controlled ovarian stimulation. Vasc. Cell.

[35] Son Y., Cox J.M., Stevenson J.L., Cooper J.A., Paton C.M. (2020). Angiopoietin-1 protects 3T3-L1 preadipocytes from saturated fatty acid-induced cell death. Nutr. Res..

[36] Di Pietro M., Pascuali N., Parborell F., Abramovich D. (2018). Ovarian angiogenesis in polycystic ovary syndrome. Reproduction.

[37] Wang B., Hao M., Yang Q., Li J., Guo Y. (2016). Follicular fluid soluble receptor for advanced glycation endproducts (sRAGE): A potential protective role in polycystic ovary syndrome. J. Assist. Reprod. Genet..

[38] Steenbeke M., De Bruyne S., De Buyzere M., Lapauw B., Speeckaert R., Petrovic M., Delanghe J.R., Speeckaert M.M. (2021). The role of soluble receptor for advanced glycation end-products (sRAGE) in the general population and patients with diabetes mellitus with a focus on renal function and overall outcome. Crit. Rev. Clin. Lab. Sci..

[39] Mouanness M., Nava H., Dagher C., Merhi Z. (2022). Contribution of Advanced Glycation End Products to PCOS Key Elements: A Narrative Review. Nutrients.

[40] Pertynska-Marczewska M., Diamanti-Kandarakis E., Zhang J., Merhi Z. (2015). Advanced glycation end products: A link between metabolic and endothelial dysfunction in polycystic ovary syndrome?. Metabolism.

[41] van Houten E.L., Laven J.S., Louwers Y.V., McLuskey A., Themmen A.P., Visser J.A. (2013). Bone morphogenetic proteins and the polycystic ovary syndrome. J. Ovarian Res..

[42] Shimasaki S., Moore R.K., Erickson G.F., Otsuka F. (2003). The role of bone morphogenetic proteins in ovarian function. Reprod. Suppl..

[43] Khalaf M., Morera J., Bourret A., Reznik Y., Denoual C., Herlicoviez M., Mittre H., Benhaim A. (2013). BMP system expression in GCs from polycystic ovary syndrome women and the in vitro effects of BMP4, BMP6, and BMP7 on GC steroidogenesis. Eur. J. Endocrinol..

[44] Nautiyal H., Imam S.S., Alshehri S., Ghoneim M.M., Afzal M., Alzarea S.I., Guven E., Al-Abbasi F.A., Kazmi I. (2022). Polycystic Ovarian Syndrome: A Complex Disease with a Genetics Approach. Biomedicines.

[45] Levet S., Ouarne M., Ciais D., Coutton C., Subileau M., Mallet C., Ricard N., Bidart M., Debillon T., Faravelli F. (2015). BMP9 and BMP10 are necessary for proper closure of the ductus arteriosus. Proc. Natl. Acad. Sci. USA.

[46] Liu R., Hu W., Li X., Pu D., Yang G., Liu H., Tan M., Zhu D. (2019). Association of circulating BMP9 with coronary heart disease and hypertension in Chinese populations. BMC Cardiovasc. Disord..

[47] Murri M., Luque-Ramírez M., Insenser M., Ojeda-Ojeda M., Escobar-Morreale H.F. (2013). Circulating markers of oxidative stress and polycystic ovary syndrome (PCOS): A systematic review and meta-analysis. Hum. Reprod. Update.

[48] Jeelani H., Ganie M.A., Masood A., Amin S., Kawa I.A., Fatima Q., Manzoor S., Parvez T., Naikoo N.A., Rashid F. (2019). Assessment of PON1 activity and circulating TF levels in relation to BMI, testosterone, HOMA-IR, HDL-C, LDL-C, CHO, SOD activity and TAC in women with PCOS: An observational study. Diabetes Metab. Syndr..

[49] Sabuncu T., Vural H., Harma M., Harma M. (2001). Oxidative stress in polycystic ovary syndrome and its contribution to the risk of cardiovascular disease. Clin. Biochem..

[50] Masjedi F., Keshtgar S., Agah F., Karbalaei N. (2019). Association Between Sex Steroids and Oxidative Status with Vitamin D Levels in Follicular Fluid of Non-obese PCOS and Healthy Women. J. Reprod. Infertil..

[51] Liu M., Sun X., Chen B., Dai R., Xi Z., Xu H. (2022). Insights into Manganese Superoxide Dismutase and Human Diseases. Int. J. Mol. Sci..

[52] Duica F., Danila C.A., Boboc A.E., Antoniadis P., Condrat C.E., Onciul S., Suciu N., Cretoiu S.M., Varlas V.N., Cretoiu D. (2021). Impact of Increased Oxidative Stress on Cardiovascular Diseases in Women with Polycystic Ovary Syndrome. Front. Endocrinol..

[53] Reindel R., Bischof J., Kim K.Y., Orenstein J.M., Soares M.B., Baker S.C., Shulman S.T., Perlman E.J., Lingen M.W., Pink A.J. (2014). CD84 is markedly up-regulated in Kawasaki disease arteriopathy. Clin. Exp. Immunol..

[54] Lei Y., Takahama Y. (2012). XCL1 and XCR1 in the immune system. Microbes Infect..

[55] Chen Y., Nilsson A.H., Goncalves I., Edsfeldt A., Engstrom G., Melander O., Orho-Melander M., Rauch U., Tengryd C., Venuraju S.M. (2020). Evidence for a protective role of placental growth factor in cardiovascular disease. Sci. Transl. Med..

[56] Palpant N.J., Bedada F.B., Peacock B., Blazar B.R., Metzger J.M., Tolar J. (2011). Cardiac disease in mucopolysaccharidosis type I attributed to catecholaminergic and hemodynamic deficiencies. Am. J. Physiol. Heart Circ. Physiol..

[57] Alawo D.O.A., Tahir T.A., Fischer M., Bates D.G., Amirova S.R., Brindle N.P.J. (2017). Regulation of Angiopoietin Signalling by Soluble Tie2 Ectodomain and Engineered Ligand Trap. Sci. Rep..

[58] Scotti L., Parborell F., Irusta G., De Zuniga I., Bisioli C., Pettorossi H., Tesone M., Abramovich D. (2014). Platelet-derived growth factor BB and DD and angiopoietin1 are altered in follicular fluid from polycystic ovary syndrome patients. Mol. Reprod. Dev..

[59] Amor M., Moreno Viedma V., Sarabi A., Grun N.G., Itariu B., Leitner L., Steiner I., Bilban M., Kodama K., Butte A.J. (2016). Identification of matrix metalloproteinase-12 as a candidate molecule for prevention and treatment of cardiometabolic disease. Mol. Med..

[60] Wang C.Y., Zhang C.P., Li B.J., Jiang S.S., He W.H., Long S.Y., Tian Y. (2020). MMP-12 as a potential biomarker to forecast ischemic stroke in obese patients. Med. Hypotheses.

[61] Piani F., Tossetta G., Fantone S., Agostinis C., Di Simone N., Mandala M., Bulla R., Marzioni D., Borghi C. (2023). First Trimester CD93 as a Novel Marker of Preeclampsia and Its Complications: A Pilot Study. High. Blood Press. Cardiovasc. Prev..

[62] Witkowski M., Landmesser U., Rauch U. (2016). Tissue factor as a link between inflammation and coagulation. Trends Cardiovasc. Med..

[63] Gonzalez F., Kirwan J.P., Rote N.S., Minium J. (2013). Elevated circulating levels of tissue factor in polycystic ovary syndrome. Clin. Appl. Thromb. Hemost..

[64] Fukui K., Yamada H., Matsubara H. (2012). [Pathophysiological role of tissue renin-angiotensin-aldosterone system (RAAS) in human atherosclerosis]. Nihon Rinsho.

[65] Arefi S., Mottaghi S., Sharifi A.M. (2013). Studying the correlation of renin-angiotensin-system (RAS) components and insulin resistance in polycystic ovary syndrome (PCOs). Gynecol. Endocrinol..

[66] Mejia-Montilla J., Reyna-Villasmil E., Torres-Cepeda D., Santos-Bolivar J., Reyna-Villasmil N., Bravo-Henriquez A. (2012). [Plasma renin and aldosterone levels in obese and non-obese women with polycystic ovary syndrome]. Endocrinol. Nutr..

[67] Moin A.S.M., Sathyapalan T., Atkin S.L., Butler A.E. (2020). Renin-Angiotensin System overactivation in polycystic ovary syndrome, a risk for SARS-CoV-2 infection?. Metabol. Open.

[68] Connolly A., Leblanc S., Baillargeon J.P. (2018). Role of Lipotoxicity and Contribution of the Renin-Angiotensin System in the Development of Polycystic Ovary Syndrome. Int. J. Endocrinol..

[69] Gonzalez F. (2012). Inflammation in Polycystic Ovary Syndrome: Underpinning of insulin resistance and ovarian dysfunction. Steroids.

[70] Obradovic M., Sudar-Milovanovic E., Soskic S., Essack M., Arya S., Stewart A.J., Gojobori T., Isenovic E.R. (2021). Leptin and Obesity: Role and Clinical Implication. Front. Endocrinol..

[71] Fruhbeck G., Catalan V., Ramirez B., Valenti V., Becerril S., Rodriguez A., Moncada R., Baixauli J., Silva C., Escalada J. (2022). Serum Levels of IL-1 RA Increase with Obesity and Type 2 Diabetes in Relation to Adipose Tissue Dysfunction and are Reduced After Bariatric Surgery in Parallel to Adiposity. J. Inflamm. Res..

[72] Lana J.P., de Oliveira M.C., Silveira A.L.M., Yamada L.T.P., Costa K.A., da Silva S.V., de Assis-Ferreira A., Gautier E.L., Dussaud S., Pinho V. (2024). Role of IL-18 in adipose tissue remodeling and metabolic dysfunction. Int. J. Obes..

[73] Harakeh S., Kalamegam G., Pushparaj P.N., Al-Hejin A., Alfadul S.M., Al Amri T., Barnawi S., Al Sadoun H., Mirza A.A., Azhar E. (2020). Chemokines and their association with body mass index among healthy Saudis. Saudi J. Biol. Sci..

[74] Eleazu C., Omar N., Lim O.Z., Yeoh B.S., Nik Hussain N.H., Mohamed M. (2019). Obesity and Comorbidity: Could Simultaneous Targeting of esRAGE and sRAGE Be the Panacea?. Front. Physiol..

[75] Jakubiak G.K., Osadnik K., Lejawa M., Osadnik T., Golawski M., Lewandowski P., Pawlas N. (2021). “Obesity and Insulin Resistance” Is the Component of the Metabolic Syndrome Most Strongly Associated with Oxidative Stress. Antioxidants.

[76] Chen Y., Ma B., Wang X., Zha X., Sheng C., Yang P., Qu S. (2021). Potential Functions of the BMP Family in Bone, Obesity, and Glucose Metabolism. J. Diabetes Res..

[77] Dilbaz B., Ozkaya E., Cinar M., Cakir E., Dilbaz S. (2011). Cardiovascular disease risk characteristics of the main polycystic ovary syndrome phenotypes. Endocrine.

