# Supplementary material for: Cardiovascular Risk Biomarkers in Women with and Without Polycystic Ovary Syndrome
Source: Biomolecules. 2024 Dec 24;15(1):4. doi: 10.3390/biom15010004 (PMC11763313; doi:10.3390/biom15010004)
Supplement: Supplementary file 1 [file biomolecules-15-00004-s001.zip › biomolecules-3382696-supplementary.pdf]

Supplementary Table 1. Cardiovascular risk proteins that were analysed using SOMAscan

Genes	Target full name
BMP-6	Bone morphogenetic protein 6
SLAF7	SLAM family member 7
ATS13	A disintegrin and metalloproteinase with thrombospondin motifs 13
ANGPT1	Angiopoietin-1
Adrenomedullin	Adrenomedullin
ATS13	A disintegrin and metalloproteinase with thrombospondin motifs 13
SRCN1	Proto-oncogene tyrosine-protein kinase Src
IL-6	Interleukin-6
TRAIL R1	Tumor necrosis factor receptor superfamily member 10A
IDUA	Alpha-L-iduronidase
RANK	Tumor necrosis factor receptor superfamily member 11A
TRAIL R2	Tumor necrosis factor receptor superfamily member 10B
Marapsin	Serine protease 27
sTie-2	Angiopoietin-1 receptor, soluble
TF	Tissue Factor
PDGF Rb	Platelet-derived growth factor receptor beta
IL-27	Interleukin-27
Gro-a	Growth-regulated alpha protein
PIGR	Polymeric immunoglobulin receptor
sRAGE	Advanced glycosylation end product-specific receptor, soluble
MnSOD	Superoxide dismutase [Mn], mitochondrial
HGH	Somatotropin
FST	Follistatin

SLAF5	SLAM family member 5
PAPP-A	Pappalysin-1
Renin	Renin
TSP2	Thrombospondin-2
XCL1	Lymphotactin
IL-16	Interleukin-16
TARC	C-C motif chemokine 17
MMP-7	Matrilysin
Bone proteoglycan II	Decorin
DKK1	Dickkopf-related protein 1
ART	Agouti-related protein
HB-EGF	Heparin-binding EGF-like growth factor
GDF2	Growth/differentiation factor 2
MMP-12	Macrophage metalloelastase
ACE2	Angiotensin-converting enzyme 2
PD-L2	Programmed cell death 1 ligand 2
TACI	Tumor necrosis factor receptor superfamily member 13B
Leptin	Leptin
sCD4	T-cell surface glycoprotein CD4
IgE	Immunoglobulin E
FGF23	Fibroblast growth factor 23
BNP-32	Brain natriuretic peptide 32
IL-2 sRa	Interleukin-2 receptor subunit alpha
IL-1Ra	Interleukin-1 receptor antagonist protein
PlGF	Placenta growth factor
BOC	Brother of CDO
LEG9	Galectin-9
IL-18 Ra	Interleukin-18 receptor 1
FCG2B	Low affinity immunoglobulin gamma Fc region receptor II-b
Tpo	Thrombopoietin
MIP-1a	C-C motif chemokine 3
